# Quality Indicators in Palliative Radiation Oncology: Development and Pilot Testing

**DOI:** 10.1016/j.adro.2021.100856

**Published:** 2021-11-20

**Authors:** Tetsuo Saito, Naoto Shikama, Takeo Takahashi, Misako Miwa, Kazunari Miyazawa, Hitoshi Wada, Naoki Nakamura, Atsunori Yorozu, Hisayasu Nagakura, Mitsunori Miyashita

**Affiliations:** a Department of Radiation Oncology, Arao Municipal Hospital, Kumamoto, Japan; b Department of Radiology, Juntendo University School of Medicine, Tokyo, Japan; c Department of Radiation Oncology, Saitama Medical Center, Saitama Medical University, Saitama, Japan; d Department of Radiation Oncology, Sendai Kousei Hospital, Miyagi, Japan; e Department of Radiology, Showa General Hospital, Tokyo, Japan; f Department of Radiation Oncology, Southern TOHOKU Proton Therapy Center, Fukushima; g Department of Radiology, St. Marianna University School of Medicine, Kanagawa, Japan; h Department of Radiation Oncology, National Hospital Organization, Tokyo Medical Center, Tokyo, Japan; i Department of Radiology, KKR Sapporo Medical Center, Sapporo, Japan; j Department of Palliative Nursing, Health Sciences, Tohoku University Graduate School of Medicine, Miyagi, Japan

## Abstract

**Purpose:**

A quality indicator (QI) is a valuable tool to evaluate the quality of health care systems. In palliative radiation oncology, only a few related QIs have been developed to date. In this study, we sought to develop and pilot test QIs that assess the quality of care in palliative radiation therapy.

**Methods and Materials:**

A modified Delphi method was used to establish consensus with an expert panel. The panel consisted of 8 radiation oncologists who have expertise in palliative radiation oncology and 1 expert on Delphi methodology. Online panel meetings and e-mail surveys were conducted to develop QIs on palliative radiation therapy for bone and brain metastases. Feasibility of measurement was assessed though pilot surveys that were conducted by radiation oncologists at 5 facilities.

**Results:**

After 3 online meetings and 2 e-mail surveys, we developed 4 QIs on bone metastases and 3 QIs on brain metastases. Two email surveys and 2 pilot surveys confirmed the validity of QIs and the feasibility of measurement, respectively.

**Conclusions:**

We developed valid and feasible QIs on palliative radiation therapy for bone and brain metastases. Our work may contribute to reduce the evidence–practice gaps in palliative radiation oncology.

## Introduction

Clinical practice is infrequently performed in accordance with available evidence or clinical guidelines.^1^ Knowing the evidence is quite diffferent from implementing it. Difficulties in implementing evidence-based practices have been shown in palliative radiation oncology.^2^ As a first step to improve the implementation of medical practices, it is necessary to recognize and measure the evidence–practice gaps. A quality indicator (QI) is a valuable tool to evaluate the quality of health care and a basis for the continuous implementation of improvements in health care.^3^ Another role of QIs is to promote accountability in regulatory agencies or consumers.^4^ Process QIs are a widely used tool to evaluate the process invloved in health care delivery.^5^ In general, process QIs are presented as numerators and denominators (the percentage of patients for whom recommended medical care was conducted); ie, the denominator represents the number of patients for whom the QI is applicable and the numerator represents the number of patients for whom the standard of care was met.

Palliative care is a field in which QIs have been eagerly studied.^5^, ^6^, ^7^, ^8^ However, in palliative radiation therapy,^9^ only a few related QIs have been developed to date.^5^^,^^10^, ^11^, ^12^ In the present study, we developed QIs related to processes in palliative radiation therapy for bone and brain metastases.

## Methods and Materials

A modified Delphi method^13^ was used to establish consensus with an expert panel. The modified Delphi method, which consists of repeated rounds of voting, is effective in determining expert consensus even when there is little or no definitive evidence and when experts'opinions are important.^14^ Several studies have used this method to develop QIs.^6^^,^^10^^,^^13^ One panel member (N.S.) sent an e-mail to potential panel members, inviting them to participate. The panel consisted of 8 radiation oncologists with expertise in palliative radiation oncology and 1 expert on Delphi methodology.

Two of the 8 radiation oncologist panel members (N.S. and T.S. first examined and identified existing QIs and clinical guidelines on palliative radiation therapy for bone and brain metastases and then developed the initial set of potential QIs. These 2 members discussed the potential QIs’ validity and feasibility of measurement and drafted 12 candidate QIs (5 QIs on bone metastases and 7 QIs on brain metastases). For each of these candidate QIs, the 2 members developed a worksheet that described a brief title, definitions of the denominator and numerator, data source, available evidence, the importance of measurement, and the potential for the improvement of practice.

**Fig. 1:**
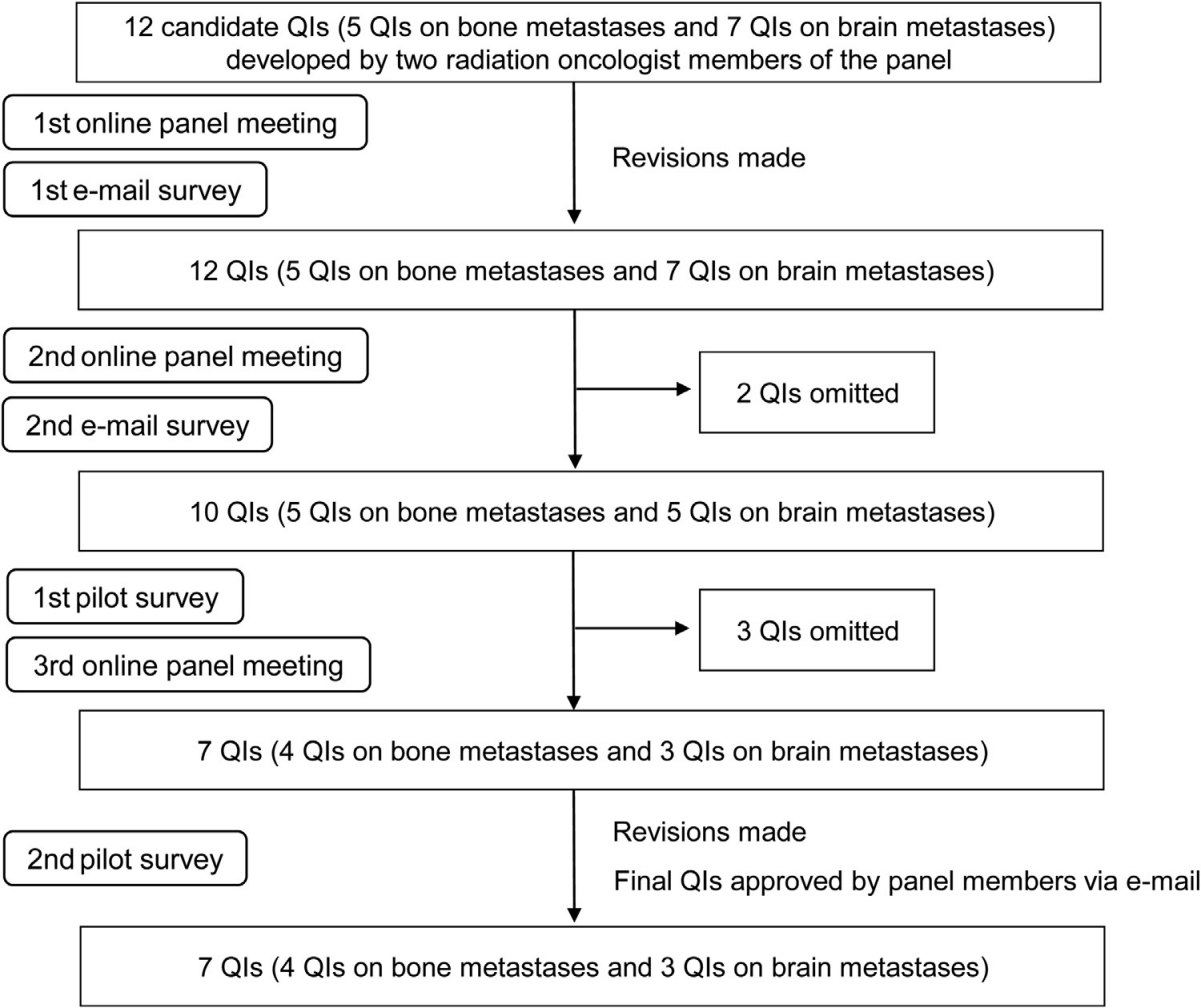
Process of the development and pilot testing of quality indicators (QIs).

Delphi rounds were subsequently performed (Fig 1). In the first online panel meeting, the method of developing and pilot testing of QIs was explained by 1 panel member (N.S.), and the validity of the 12 candidate QIs was discussed by the other members. In 2 e-mail surveys, the 8 radiation oncologist panel members were e-mailed a sheet that listed the candidate QIs with the aforementioned worksheets that helped to evaluate the QIs. The 8 radiation oncologist panel members rated the validity and feasibility of the 12 QIs using a 10-point scale (0-9) and e-mailed the rating sheets to one of the panel members (N.S.). A higher score indicated higher validity and feasibility. We set the criteria of adopting a QI as follows: for a QI to be adopted, there had to be an agreement between the ratings of the validity score of a QI (specifically, after the highest and lowest scores were excluded, the range had to be ≤2 points) and the median of the validity score had to be ≥6.5.

We performed 2 pilot surveys to assess the feasibility of the measurement of the QIs. For the pilot surveys, the 2 members (N.S. and T.S) developed manuals and forms for the measurement of the QIs to achieve a high reproducibility in measurement; the manuals provided key information on the definitions of QIs and the data source (eg, the medical record or radiology information system). The first pilot survey was performed by 5 radiation oncologists at 5 centers (3 university hospitals and 2 nonacademic centers) after receiving the approval from the participating centers’ institutional review boards (reference numbers, 20-22 [Arao Municipal Hospital]; 20-234 [Juntendo University School of Medicine]; 2483 [Saitama Medical Center], 2-48 [Sendai Kousei Hospital], 248 [Showa General Hospital]). For the denominator of each QI, we examined the data of patients who received radiation therapy between January 1, 2019, and June 30, 2019. When the denominator reached 10, the search for patients was allowed to be declared complete. The second pilot survey was performed by 4 radiation oncologists at 4 centers (2 university hospitals and 2 nonacademic centers; 1 university hospital was excluded from the 5 centers of the first pilot survey).

## Results

Two radiation oncologist members (N.S. and T.S.) of the panel drafted the initial set of 12 candidate QIs (Fig); of these, 5 were modified versions of the existing QIs^5^^,^^10^^,^^11^ and 7 were newly developed. In both e-mail surveys, all 8 radiation oncologist panel members returned the rating sheets. At the first e-mail survey, the ratings of the 8 radiation oncologist panel members allowed all 12 QIs to be retained in the list while requiring revisions to the definition of some of the QIs. These revisions were made by the 2 members (N.S. and T.S.) who first drafted the candidate QIs. After the second online panel meeting when the revised QIs were discussed, the second e-mail survey was performed, and 2 of the 12 QIs were omitted from the list based on the predetermined validity criteria.

The remaining 10 QIs (5 on bone metastases and 5 on brain metastases) were then evaluated through pilot testing. In the first pilot survey, 5 radiation oncologists at 5 centers returned the survey regarding the time required for and technical difficulties in the measurement of the 10 QIs to one of the panel members (N.S.) (Table E1 in the online supplementary material). At the third online panel meeting, the members discussed the validity and feasibility of the QIs, and the members agreed that 1 QI on bone metastases and 2 on brain metastases should be omitted from the list. After further revisions to the remaining 7 QIs (4 on bone metastases and 3 on brain metastases) were made by the 2 members (N.S. and T.S.), these QIs were tested in the second pilot survey by 4 radiation oncologists at 4 centers. After the second pilot testing, further revisions to the definition of QIs were made by the 2 members (N.S. and T.S.). The revised QIs were e-mailed to the panel members and then approved as the final version of the QIs by the members (Table 1).

## Discussion

We developed 4 QIs on bone metastases and 3 QIs on brain metastases. These QIs were considered valid and feasible through Delphi rounds and pilot surveys. The development of these QIs may contribute to palliative radiation oncology, given the paucity of existing QIs on palliative radiation therapy.^15^

QIs can be classified into structure, process, and outcome indicators.^16^^,^^17^ The structure of the health care system (eg, equipment, staff, or policy) provides a framework on which the health care process is performed, and the process of care would lead to outcomes. In this study, we developed and pilot tested QIs on the process of care. Because the quality of palliation was impossible to evaluate using medical records, we could not develop QIs for the outcome. Moreover, patients belonging to different backgrounds and treated in different facilities may have compromised the quality of the comparison of outcomes between facilities because different facilities may have different treatment policies. For example, to patients with poor performance status and brain metastases, radiation therapy may be offered in some facilities but not in others. Therefore, comparing the overall survival rates after radiation therapy for brain metastases between these facilities may be problematic. Nonetheless, we evaluated the process of care mainly based on the information from medical records and compared the results of the measurement of QIs between facilities.

QIs have to be feasible as well as valid. In the present study, we developed QIs for which the necessary information was derived from medical records or the radiology information system within a reasonable time. If acquiring information for the measurement of a QI is cumbersome, assessment of the QI in many facilities is unfeasible. However, to make a QI more feasible can make the QI less valid. It is important to balance validity and feasibility. For example, to identify patients with metastatic spinal cord compression, precise identification may be performed based on medical records and diagnostic imaging. However, to avoid too much effort in acquiring information, we decided to identify patients with spinal cord compression specifically using medical records.

Of the 7 QIs that we finally developed, 2 QIs that were on bone metastases were modified versions of existing QIs on palliative radiation therapy. Regarding our QI pertaining to the choice of radiation schedules, for a patient to be included in the numerator, the patient should receive radiation therapy in ≤10 fractions or the reason for the use of extended-fraction radiation therapy should be included in their medical record. Although this is similar to the National Quality Forum QI #1822,^11^ the National Quality Forum QI requires that fractionation should be of 30 Gy in 10 fractions, 24 Gy in 6 fractions, 20 Gy in 5 fractions, or 8 Gy in 1 fraction. Therefore, our definition may identify slightly more patients who can be included in the numerator. Moreover, we allowed the use of extended-fraction radiation therapy—considering, for example, radiation therapy for oligometastases—as long as the reason for the use of the schedule was included in the medical record.

Our QI regarding the prompt initiation of radiation therapy for metastatic spinal cord compression is similar to that developed by the Cancer Quality-ASSIST Project, which requires radiation therapy or surgical decompression to be initiated within 24 hours after spinal cord compression is confirmed through radiologic examination.^5^ Our QI requires radiation therapy to be initiated promptly after the referral to radiation oncology. This is partly because it is sometimes difficult to determine when the spinal cord compression was confirmed through radiologic examination. For example, if a bulky spinal metastasis was radiologically confirmed 2 weeks ago and paralysis began yesterday, when was the cord compression confirmed? Our QI, which estimates the promptness after the referral to radiation oncology, assesses only the quality of care provided by the radiation oncology department.

A limitation of the present study was that the pilot survey was conducted in only 5 centers; therefore, the feasibility of the measurement could not be fully evaluated. Second, our QIs included palliative radiation therapy exclusively for bone and brain metastases. Further research regarding other areas in palliative radiation oncology is necessary Table 1.

**Table 1:** Quality indicators finally developed through the modified Delphi method

			Validity*			
Brief description	Denominator	Numerator	Median	Range	Range after excluding highest and lowest scores	Agreement
Bone metastases						
Choice of radiation schedules	Patients who received radiation therapy for painful bone metastases†	Patients who received radiation therapy in ≤10 fractions or for whom the reason for the use of extended-fraction radiation therapy was written in the medical record	7.5	6-8	6-8	Yes
Assessment of pain before radiation therapy	Patients who received radiation therapy for painful bone metastases†	Patients for whom some description on pain before radiation therapy was written on the medical record	7	5-9	6-8	Yes
Prompt initiation of radiation therapy for metastatic spinal cord compression	Patients who received radiation therapy for metastatic spinal cord compression‡	Patients for whom radiation therapy was initiated on the day of referral to radiation oncology or the next day	7	6-8	7-8	Yes
Concurrent use of steroids with radiation therapy for metastatic spinal cord compression	Patients who received radiation therapy for metastatic spinal cord compression‡	Patients for whom steroids were initiated or increased concurrently with the initiation of radiation therapy	6.5	3-8	5-7	Yes
Brain metastases						
Assessment of performance status before radiation therapy	Patients who received radiation therapy for brain metastases	Patients for whom performance status before radiation therapy was recorded by radiation oncologists in the medical record or radiology information system	7.5	7-9	7-8	Yes
Completion of planned radiation therapy	Patients who received whole-brain radiation therapy for brain metastases	Patients for whom the planned radiation therapy was completed	7	6-8	7-7	Yes
Initiation of radiation therapy without delay	Patients who received whole-brain radiation therapy for brain metastases§	Patients for whom the radiation therapy was initiated within 10 days from referral to radiation oncology	7	5-9	6-8	Yes

⁎ Scores on a 10-point scale (0-9) at the second e-mail survey.

† Patients who had received radiation therapy or surgery to the same bone metastases should be excluded from the denominator.

‡ When a symptom in the lower extremities, caused by spinal cord compression, was written in the medical record or referral letter.

§ Patients who received intensity modulated whole-brain radiation therapy should be excluded from the denominator.

In summary, the developed QIs provided tools to assess the quality of the implementation of palliative radiation therapy for bone and brain metastases. This study may contribute to reducing the evidence–practice gaps in palliative radiation oncology. The QIs in this study were developed under current practices and evidence, and therefore, they must be modified to align with changes in practice patterns or emerging new evidence in the future.

## Appendix Supplementary materials

Image, application 1
