# Supplementary material for: Quality Indicators in Palliative Radiation Oncology: Development and Pilot Testing
Source: Adv Radiat Oncol. 2021 Nov 20;7(2):100856. doi: 10.1016/j.adro.2021.100856 (PMC8818916; doi:10.1016/j.adro.2021.100856)
Supplement: Supplementary file 1 [file mmc1.docx]

| **eTable 1** Results of the first pilot survey | | | | | | |
| --- | --- | --- | --- | --- | --- | --- |
| Brief description | Denominator | Numerator |  | Results of the pilot survey | | |
|  |  |  |  | Denominator | Numerator | Median time required for the survey, hour (range) |
| **Bone metastases** | | | | | | |
| Choice of radiation schedules | The patients who received radiation therapy for painful bone metastases^b^ | The patients who received radiation therapy in ≤ 10 fractions, or for whom the reason for the use of extended-fraction radiation therapy was written in the medical chart |  | 50 | 46 | 0.5 (0.3–1.0) |
| Single-fraction therapy for patients with poor survival^a^ | The patients for whom radiotherapy for bone metastases was initiated within two months before death | The patient for whom the radiation schedule was 8 Gy in 1 fraction |  | 23 | 4 | 1 (0.4–2) |
| Assessment of pain before radiation therapy | The patients who received radiation therapy for painful bone metastases^b^ | The patients for whom some description on pain before radiation therapy was written on the medical chart |  | 60 | 52 | 0.5 (0.3–1.0) |
| Prompt initiation of radiation therapy for metastatic spinal cord compression | The patients who received radiation therapy for metastatic spinal cord compression^c^ | The patients for whom radiation therapy was initiated on the day of referral to radiation oncology or the next day |  | 26 | 19 | 0.5 (0.4–2) |
| Concurrent use of steroids with radiation therapy for metastatic spinal cord compression | The patients who received radiation therapy for metastatic spinal cord compression^c^ | The patients for whom dexamethasone or betamethasone of ≥ 10 mg were used concurrently with the radiation therapy |  | 24 | 3 | 1 (0.3–2) |
| **Brain metastases** | | | | | | |
| Assessment of performance status before radiation therapy | The patients who were referred to radiation oncology for radiation therapy for brain metastases | The patients for whom performance status before radiation therapy was recorded by radiation oncologists in the medical chart |  | 51 | 29 | 0.4 (0.1–1) |
| Completion of planned radiation therapy | The patients for whom whole brain radiation therapy for brain metastases in > 10 fractions were planned | The patients for whom the planned radiation therapy was not completed |  | 5 | 0 | 0.5 (0.3–1) |
| Initiation of radiation therapy without delay | The patients who received whole brain radiation therapy for brain metastases^d^ | The patients for whom the radiation therapy was not initiated within ten days from referral to radiation oncology |  | 40 | 3 | 0.3 (0.1–1) |
| Implementation of radiation therapy for patients with poor performance status^a^ | The patients who received radiation therapy for brain metastases | The patients with Eastern Cooperative Oncology Group performance status of 3–4 |  | 51 | 11 | 0.5 (0.3–1) |
| Extended-field radiation therapy for patients with poor performance status^a^ | The patients who received whole brain radiation therapy for brain metastases and whose Eastern Cooperative Oncology Group performance status was 3–4 | The patients for whom the planned number of fraction was > 10 |  | 10 | 0 | 0.3 (0.1–1) |
| The part of the definitions of quality indicators for which some modification was added after the pilot survey are shown with underline.  ^a^ Quality indicators which were excluded from the final approved set.  ^b^ Patients who had received radiation therapy or surgery to the same bone metastases should be excluded from the denominator.  ^c^ When some symptom in the lower extremities, which was caused by spinal cord compression, was written in the medical chart or referral letter. | | | | | | |
